# Robot-assisted, laparoscopic and open radical cystectomy for bladder cancer: A systematic review and network meta-analysis

**DOI:** 10.1590/S1677-5538.IBJU.2024.0191

**Published:** 2024-08-20

**Authors:** Zhanpo Yang, Xinmeng Dou, Wenhui Zhou, Qian Liu

**Affiliations:** 1 Tianjin First Central Hospital Department of Urology Tianjin China Department of Urology, Tianjin First Central Hospital, Tianjin, China

**Keywords:** Urinary Bladder Neoplasms, Robotic Surgical Procedures, Laparoscopy

## Abstract

**Objectives:**

To evaluate the safety and effectiveness of robot-assisted radical cystectomy (RARC), laparoscopic radical cystectomy (LRC), and open radical cystectomy (ORC) in bladder cancer.

**Methods:**

A literature search for network meta-analysis was conducted using international databases up to February 29, 2024. Outcomes of interest included baseline characteristics, perioperative outcomes and oncological outcomes.

**Results:**

Forty articles were finally selected for inclusion in the network meta-analysis. Both LRC and RARC were associated with longer operative time, smaller amount of estimated blood loss, lower transfusion rate, shorter time to regular diet, fewer incidences of complications, and fewer positive surgical margin compared to ORC. LRC had a shorter time to flatus than ORC, while no difference between RARC and ORC was observed. Considering lymph node yield, there were no differences among LRC, RARC and ORC. In addition, there were statistically significant lower transfusion rates (OR=-0.15, 95% CI=-0.47 to 0.17), fewer overall complication rates (OR=-0.39, 95% CI=-0.79 to 0.00), fewer minor complication rates (OR=-0.23, 95% CI=-0.48 to 0.02), fewer major complication rates (OR=-0.23, 95% CI=-0.68 to 0.21), fewer positive surgical margin rates (OR=0.22, 95% CI=-0.27 to 0.68) in RARC group compared with LRC group.

**Conclusion:**

LRC and RARC could be considered as a feasible and safe alternative to ORC for bladder cancer. Notably, compared with LRC, RARC may benefit from significantly lower transfusion rates, fewer complications and lower positive surgical margin rates. These data thus showed that RARC might improve the management of patients with muscle invasive or high-risk non-muscle invasive bladder cancer.

## INTRODUCTION

Bladder cancer is the 10th most common malignancy in the World, accounting for approximately 573,000 new cases and 213,000 deaths in 2020 (1). The incidence and mortality rate of bladder cancer in men is about 4 times that of women. According to the classification of invasion depth, bladder cancer can be divided into non-muscle-invasive bladder cancer (NMIBC) and muscle-invasive bladder cancer (MIBC) (2). Approximately 75% of new cases are diagnosed as NMIBC, and 25% present as MIBC. Unfortunately, approximately 40% of NMIBC patients eventually progress to MIBC (3).

Currently, open radical cystectomy (ORC) is still the standard surgical treatment for patients with MIBC or high-risk of NMIBC (4), which can effectively achieve local control of the tumor and long-term disease-free survival (5, 6). However, ORC is associated with a high postoperative morbidity, such as urinary tract infection, urinary leak, renal failure, ileus and thromboembolic complications. Previous research data show that the incidence of postoperative complications after ORC is as high as 40% to 60%, even if the surgeon knows enough about pelvic anatomy and the surgical technique is continuously improved (7).

Recently, with the development of minimally invasive technology, laparoscopic radical cystectomy (LRC) and robotic assisted radical cystectomy (RARC) have become new methods of treating bladder cancer and are gradually being promoted (8, 9). Compared to LRC, RARC has technological superiorities of better visibility, improved degrees of freedom, and lower learning curves, which helps to overcome the technical difficulties of LRC, including operator fatigue, tremor, and internal suturing. Nevertheless, the cost of RARC is much higher than that LRC, which remains a common alternative to ORC in many medical centers (10).

There is limited evidence comparing RARC, LRC and ORC for bladder cancer. Dong et al. (11) compared long-term oncologic outcomes of three surgical methods but didn't include perioperative outcomes. Kowalewski et al. (12) identified ten randomized controlled trials that compared RARC, LRC and ORC, the results showed that no differences in overall survival and recurrence-free survival between RARC and ORC, with moderate certainty of evidence. These studies had small sample sizes and low levels of probative medical evidence. Therefore, we aimed to undertake a contemporary up-to-date systematic review and network meta-analysis to compare RARC, LRC and ORC for bladder cancer. The primary outcomes of this review were total operative time, estimated blood loss (EBL), intraoperative blood transfusion rate; length of hospital stays (LOS), days to regular diet, time to flatus and complications. The secondary outcomes were positive surgical margin (PSM) and lymph node yield.

## MATERIALS AND METHODS

This systematic review and meta-analysis protocol was registered with the PROSPERO International Prospective Register of Systematic Reviews (PROSPERO) (registration number: CRD42024547617).

### Evidence acquisition

The systematic review and network meta-analysis is reported in accordance with the Preferred Reporting Items for Systematic Reviews and Meta-Analyses (PRISMA) statements (13). Ethical approval was unnecessary in this study, because it was a meta-analysis of existing articles, and no individual patient data were handled.

### Literature search

A systematic search was performed in electronic databases, including PubMed, Embase, Ovid, Cochrane library and Clinical Trials.gov. The search terms were as follows: "bladder cancer", "cystectomy", "robot", "robotic", "laparoscopic", "RARC", "LRC", "ORC" and their synonyms or similar words. The searches were conducted without date restriction, from database inception to February 29, 2024, and limited to English-language articles in human adults. In addition, reference lists of all included articles and relevant reviews were searched manually to prevent missing articles. The literature search was done independently by two investigators and was resolved by discussing with the third investigator when the search results were inconsistent.

### Inclusion and exclusion criteria

Inclusion criteria: (1) patients with bladder cancer; (2) comparing at least two of three different approaches (open, laparoscopic or robot-assisted radical cystectomy); (3) the study provided analyzable data of interest: total operative time, estimated blood loss (EBL), intraoperative blood transfusion rate, length of hospital stays (LOS), days to regular diet, time to flatus, complication rate, positive surgical margin (PSM) and lymph node yield; (4) whole text was accessible.

Conference abstracts, review articles, editorials, comments, and letters to the editor were excluded.

### Study selection and Data extraction

The detailed data were as follows: (1) first author's name and publication time; (2) study design; (3) treatment and sample size; (4) patient characteristics (gender ratio and age distribution); (5) perioperative outcomes: total operative time, estimated blood loss (EBL), intraoperative blood transfusion rate; length of hospital stays (LOS), days to regular diet, time to flatus and 90-day postoperative complication (stratified by Clavien-Dindo classification (14) into all, minor [grade 1–2] and major [grade 3–5] complications); (6) oncological outcomes: positive surgical margin (PSM), lymph node yield.

### Risk of bias assessment

Two investigators independently assessed the methodological quality of articles using the Cochrane Risk of Bias Assessment Tool (15). These studies were classified into three degrees: low risk of bias, middle-risk of bias, or high risk of bias. The writers came to an agreement on certain points where they disagreed.

## Statistical analysis

Means and standard deviations (SDs) or medians and interquartile ranges (IQRs) were utilized for continuous variables. All median and IQR values were transformed to means and SDs through the methodology described by Hozo et al. (16).

Statistical analyses were performed using Review Manager (Version 5.4, Cochrane Collaboration, Oxford, UK) and Stata software (version 14.0, Stata Corporation LLC). Binary variable data are combined with relative risk (RR) or relative odds ratio (OR) statistical measures, and the 95% confidence interval (95% CI) is calculated. Continuous variables are represented by standardized mean difference (SMD) or mean difference (MD), and the 95% CI is calculated. We generated league tables and rankograms based on surface under the cumulative ranking (SUCRA) values.

## RESULTS

### Literature search results

**Figure 1:**
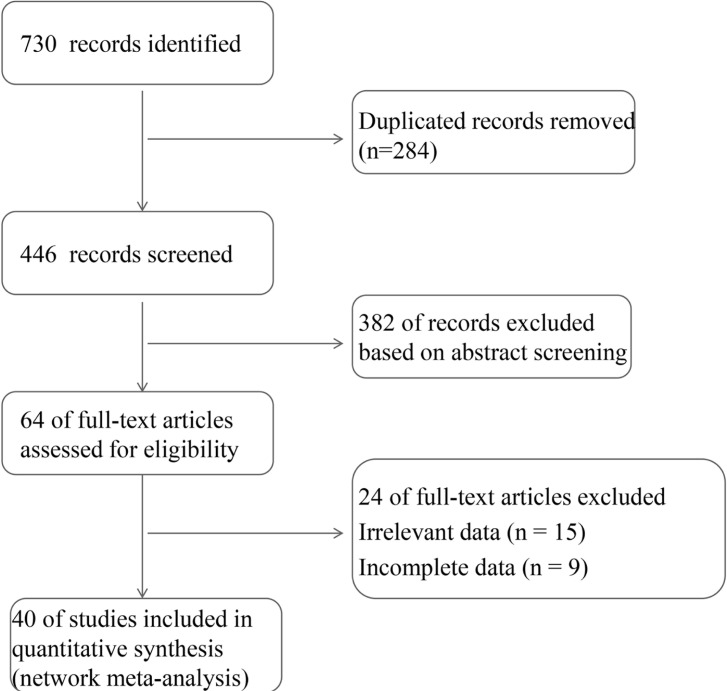
The flow diagram about the study retrieval process.

Totally of 730 relevant articles were retrieved according to the customized search strategy, 284 repeatedly published and cross-published were removed. Furthermore, 382 articles were excluded by evaluating the title and abstract. After the remaining 64 articles were searched for full text, reading, and quality assessment, twenty-four studies were excluded for the following: irrelevant data (n=15); incomplete data (n=9). Finally, 40 (3, 8, 17–53) articles were eventually included in this network meta-analysis (Figure-1), including ten RCTs, seventeen prospective articles, and twelve retrospective studies, and one case control study.

### Characteristics and risk of bias of the included studies

The basic information of the included studies is presented in Table-1. The oldest study was published in 2006 and the most updated in 2024. A total of 7156 cases were analyzed, with 2625 (37.1%) in RARC group, 924 (12.9%) in the LRC arm and 3580 (50%) in ORC arm. Median age ranged between 60 and 70 years old.

**Table 1:** Main characteristics of the studies included in network meta-analysis.

Included studies	Studies design	Treatment 1	Treatment 2	Treatment 3	Sample size	Age, years	Sex(male/ female)
Abraham et al. 2007 (17)	Prospective study	RARC	LRC	/	14/20	76.5/77.6	/
Arora et al. 2020 (18)	Retrospective study	RARC	LRC	/	188/112	68/67	168:20/92:20
Bai et al. 2021 (19)	Retrospective study	RARC	LRC	/	136/82	62.6/61	101:35/65:17
Bochner et al. 2015 (20)	RCT	RARC	/	ORC	60/58	66/65	51:9/42:16
Borghesi et al. 2018 (21)	Prospective study	RARC	/	ORC	17/33	72/72	/
Catto et al. 2022 (22)	RCT	RARC	/	ORC	161/156	69.3/68.7	128:33/122:34
Chen et al. 2017 (61)	RCT	/	LRC	ORC	29/28	78/77	20:9/19:9
Chow et al. 2018 (23)	Prospective study	RARC	/	ORC	26/13	70/75	21:5/10:3
Dixon et al. 2023 (24)	RCT	RARC	/	ORC	157/148	/	/
Galich et al. 2006 (25)	Retrospective study	RARC	/	ORC	13/24	70/70.5	10:3/18:6
Gan et al. 2013 (26)	Prospective study	RARC	LRC	ORC	20/20/19	/	/
Gastecka et al. 2018 (62)	Retrospective study	RARC	LRC	/	52/37	67/66	40:12/33:4
Guillotreau et al. 2009 (63)	Prospective study	/	LRC	ORC	38/30	67.9/64.9	36:2/25:5
Kader et al. 2013 (28)	Retrospective study	RARC	/	ORC	103/100	67/66	74:29/73:27
Khan et al. 2012 (29)	Prospective study	RARC	LRC	ORC	48/58/52	66.5/69.8/65	41:7/54:4/40:12
Khan et al. 2016 (30)	RCT	RARC	LRC	ORC	20/19/20	68.6/68.6/66.6	17:3/15:5/18:2
Kim et al. 2016 (31)	Retrospective study	RARC	LRC	ORC	58/22/150	61.5/65/68	54:4/20:2/123:27
Lin et al. 2014 (32)	RCT	/	LRC	ORC	35/35	63.2/63.6	32:3/32:3
Lisinski et al. 2022 (33)	Prospective study	/	LRC	ORC	77/82	66/65	62:15/62:20
Maibom et al. 2022 (34)	RCT	RARC	/	ORC	25/25	70/67	20:5/18:7
Mastroianni et al. 2022 (35)	RCT	RARC	/	ORC	58/58	64/66	44:14/40:18
Matsumoto et al. 2019 (36)	Retrospective study	RARC	LRC	ORC	10 10 16	67.3/67/69.2	8:2/8:2/11:5
Messer et al. 2014 (37)	Prospective study	RARC	/	ORC	20/20	69.5/64.5	18:2/16:4
Ng et al. 2010 (38)	Prospective study	RARC	/	ORC	83/104	70.9/67.2	65:18/73:31
Nix et al. 2010 (39)	RCT	RARC	/	ORC	21/20	67.4/69.2	14:7/17:3
Panwar et al. 2018 (40)	Prospective study	RARC	LRC	ORC	24/5/54	57/54/58	/
Parekh et al. 2018 (42)	RCT	RARC	/	ORC	150/152	70/67	126:24/128:24
Porpiglia et al. 2007 (43)	Prospective study	/	LRC	ORC	20/22	63.5/71	19:1/20:2
Porreca et al. 2022 (8)	Prospective study	RARC	LRC	ORC	368/46/1009	67/76/72	314:54/39:7/ 803:206
Ram et al. 2018 (44)	Prospective study	RARC	/	ORC	125/45	61.76/60.07	109:16/40:5
Rhee et al. 2006 (45)	Prospective study	RARC	/	ORC	7/23	60/67	6:1/14:9
Sharma et al. 2017 (46)	Prospective study	RARC	/	ORC	65/407	70.9/70.2	63:2/298:109
Styn et al. 2012 (64)	Retrospective study	RARC	/	ORC	50/100	66.6/65.6	□
Styn et al. 2019 (47)	Retrospective study	RARC	LRC	/	189/126	62/62.6	160:29/64:62
Tan et al. 2018 (48)	Prospective study	RARC	/	ORC	45/50	65.0/62.8	32:13/36:14
Teishima et al. 2014 (49)	Prospective study	RARC	LRC	/	6/5	68.7/67.3	/
Wang et al. 2008 (51)	Case control study	RARC	/	ORC	33/21	70/66	29:4/13:8
Yang et al. 2024 (52)	Retrospective study	RARC	/	ORC	128/461	71/70	102:26/351:110
Zhang et al. 2020 (53)	Retrospective study	RARC	LRC	/	172/126	68.1/66.2	147:25/103:23
Zhou et al. 2023 (3)	Retrospective study	/	LRC	ORC	45/45	65.5/65.3	21:24/22:23

The risk of bias according to the Cochrane Collaboration's tool ranged from intermediate to low.

**Figure 2:**
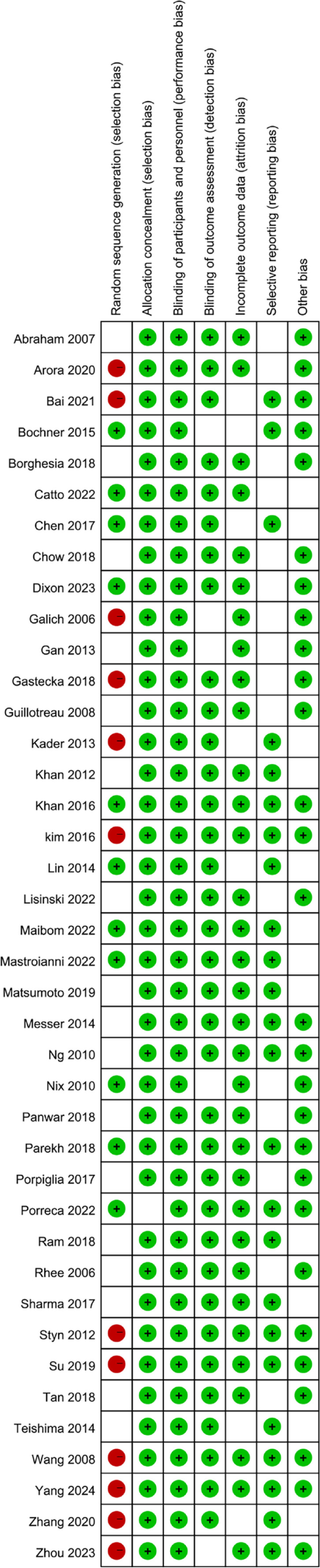
Risk of bias assessment.

The protocols and methods of all included studies were reviewed according to the Cochrane Collaboration's tool, and generally considered to have an overall low risk of bias with adequate randomization (Figure-2). Due to the physical component of surgery, blinding was not attempted in most studies. Thus, most studies were deemed at high risk of performance bias.

### Perioperative outcomes

#### Total operative time

**Figure 3:**
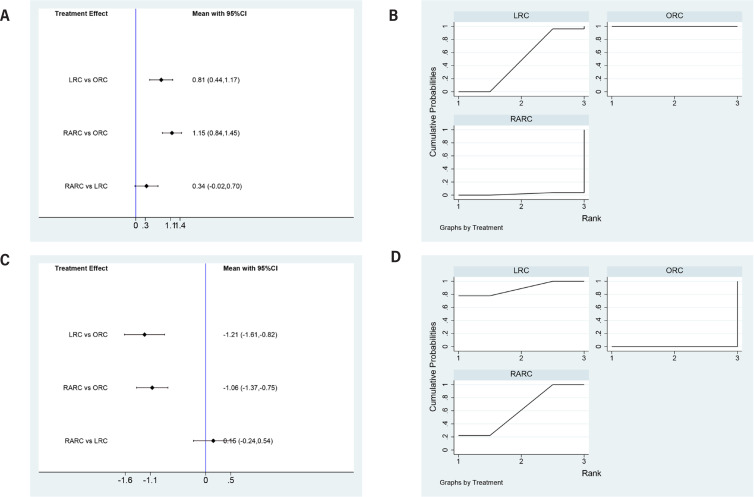
Forest plots and surface under the cumulative ranking (SUCRA) plots summarizing the meta-analyses between LRC, RARC and ORC for: (A) (B) Operative time; (C)(D) Estimated blood loss; (E)(F) Transfusion rate.

Both LRC (SMD=0.81, 95% CI=0.44 to 1.17) and RARC (SMD=1.15, 95% CI=0.84 to 1.45) had significantly longer operative time compared to ORC. No statistically difference between LRC and RARC (SMD=0.34, 95% CI=-0.02 to 0.7) (Figure-3A). Concerning SUCRA results, ORC ranked first in operative time, followed by LRC, RARC (Figure-3B), this means that RARC has the longest surgical time, followed by LRC, and ORC.

### Estimated blood loss and transfusion rate

Compared to ORC, the amount of blood loss during LRC (SMD=-1.21, 95% CI=-1.61 to −0.82) and RARC (SMD=-1.06, 95% CI=-1.37 to −0.75) was reduced at a statistically significant level. No statistically significant difference in blood loss between LRC and RARC (SMD=0.15, 95% CI=-0.24 to 0.54) was observed (Figure-3C). Concerning SUCRA results, LRC ranked first in estimated blood loss, followed by RARC, ORC (Figure-3D), this means that LRC has the least bleeding volume, followed by RARC, ORC.

**Figure 4:**
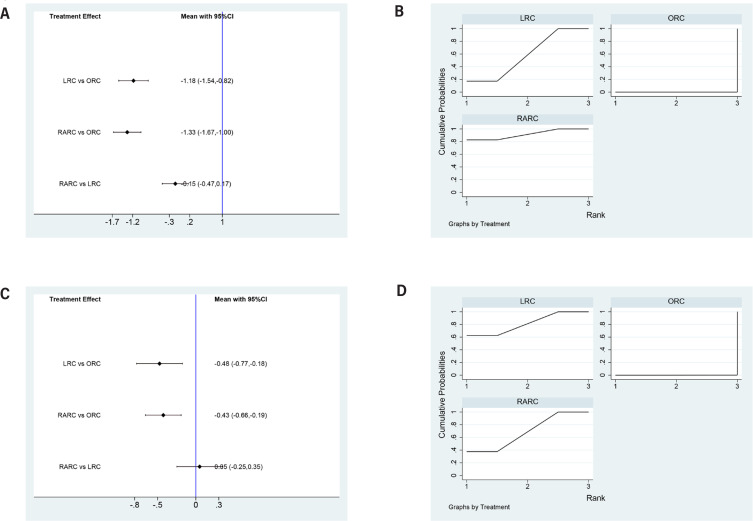
Forest plots and surface under the cumulative ranking (SUCRA) plots summarizing the meta-analyses between LRC, RARC and ORC for: (A) (B) transfusion rate; (C)(D) length of hospital stays (LOS).

Both LRC (OR=-1.18, 95% CI=-1.54 to −0.82) and RARC (OR=-1.33, 95% CI=-1.67 to −1.00) had statistically fewer transfusion rates compared to ORC. Besides, RARC had statistically fewer transfusion rates than LRC (OR=-0.15, 95% CI=-0.47 to 0.17) (Figures-4A and B).

### Length of hospital stays (LOS)

LRC (SMD=-0.48, 95% CI=-0.77 to −0.18) and RARC (SMD=-0.43, 95% CI=-0.66 to −0.19) had a shorter hospital day than ORC. No statistically significant difference in hospital stays between LRC and RARC (SMD=0.05, 95% CI=-0.245 to 0.35) was observed (Figure-4C). Concerning SUCRA results, LRC ranked first in operative time, followed by RARC, LRC (Figure-4D), this means that LRC has the shortest length of stay, followed by RARC, ORC.

### Days to regular diet

**Figure 5:**
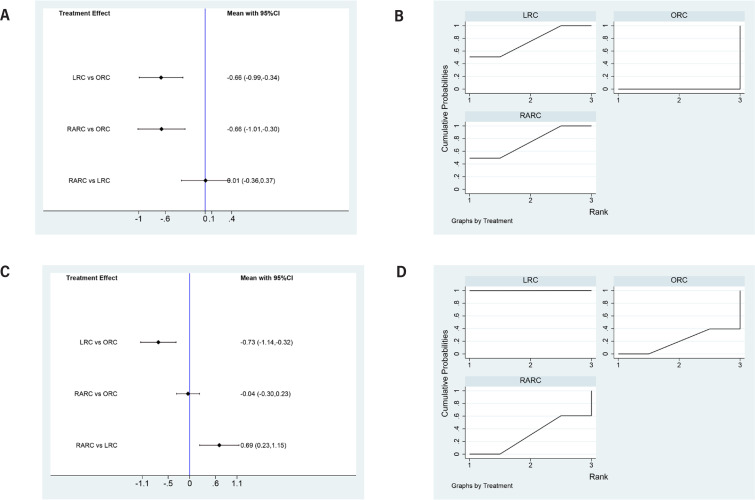
Forest plots and surface under the cumulative ranking (SUCRA) plots summarizing the meta-analyses between LRC, RARC and ORC for: (A) (B) days to regular diet; (C)(D) time to flatus.

LRC (SMD=-0.66, 95% CI=-0.99 to −0.34) and RARC (SMD=-0.66, 95% CI=-1.01 to −0.3) had a significant shorter time to regular diet than ORC. No statistically significant difference in time to regular diet between LRC and RARC was observed (SMD=0.01, 95% CI=-0.36 to 0.37) (Figure-5A). Concerning SUCRA results, ORC ranked first in operative time, followed by LR, RARC (Figure-5B), this means that RARC has the shortest time to restore normal diet, followed by LRC, ORC.

### Time to flatus

LRC (SMD=-73, 95% CI=-1.44 to −0.32) had a shorter time to flatus than ORC. No statistically significant difference in time to flatus between RARC and ORC was observed (SMD=-0.04, 95% CI=-0.3 to 0.23) (Figure-5C). Concerning SUCRA results, LRC ranked first in operative time, followed by RARC, ORC (Figure-5D), this means that LRC has the shortest time to flatus, followed by LRC, ORC.

### Complication rates

**Figure 6:**
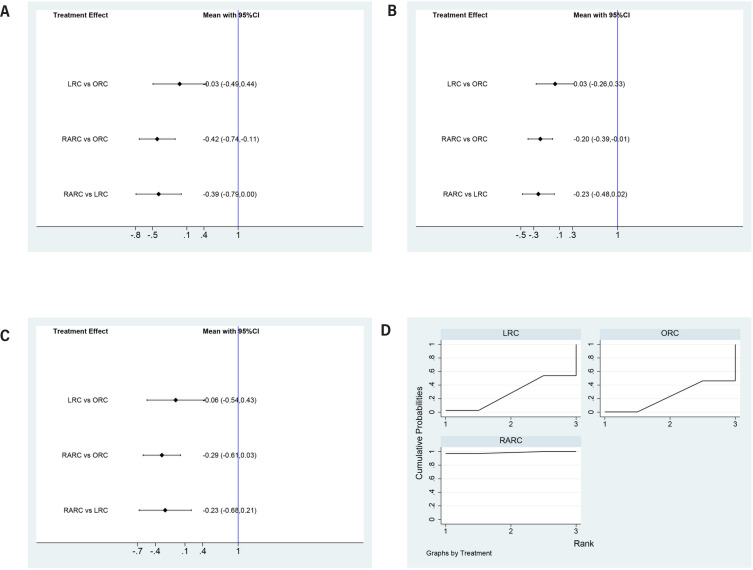
Forest plots summarizing the meta-analyses between LRC, RARC and ORC for: (A) overall complication rates; (B) minor complication rates; (C) major complication rates. (D) surface under the cumulative ranking (SUCRA) plots.

Both LRC (OR=-0.03, 95% CI=-0.49 to 0.44) and RARC (OR=-0.42, 95% CI=-0.74 to −0.11) had statistically fewer incidences of overall complications within 90 days compared to ORC. Besides, RARC had statistically fewer overall complication rates than LRC (OR=-0.39, 95% CI=-0.79 to 0.00) (Figure-6A). Similarly, LRC and RARC had statistically lower minor complication rates (LRC: OR=0.03, 95% CI=-0.26 to 0.33 and RARC: OR=-0.2, 95% CI=-0.39 to −0.01) and major complication rates (LRC: OR=0.06, 95% CI=-0.254 to 0.43 and RARC: OR=-0.29, 95% CI=-0.61 to 0.03) compared to ORC. Besides, RARC had statistically lower minor complication rates (OR=-0.23, 95% CI=-0.48 to 0.02) and major complication rates (OR=-0.23, 95% CI=-0.68 to 0.21) than LRC (Figures-6B and C). Concerning SUCRA results, RARC ranked first in complication rates, followed by LRC, ORC (Figure-6D), this means that RARC has the fewest complications, followed by LRC, ORC.

### Oncological outcomes

#### Lymph node yield

**Figure 7:**
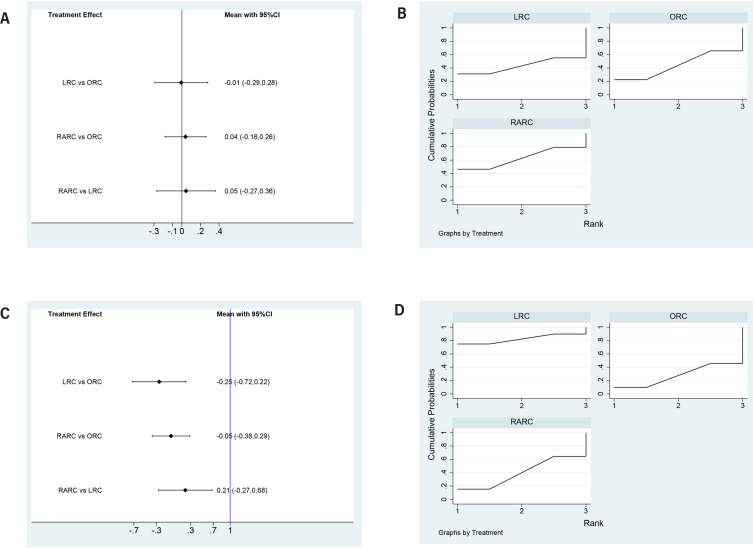
Forest plots and surface under the cumulative ranking (SUCRA) plots summarizing the meta-analyses between LRC, RARC and ORC for: (A) (B) lymph node yield; (C)(D) positive surgical margin rates.

No differences in lymph node yield were found for LRC versus ORC (SMD=-0.01, 95% CI=-0.29 to 0.28), RARC versus ORC (SMD=0.04, 95% CI=-0.18 to 0.26), and RARC versus LRC (SMD=0.05, 95% CI=-0.27 to 0.36) (Figure-7A). Concerning SUCRA results, RARC ranked first in lymph node yield, followed by LRC, ORC (Figure-7B), this means that RARC has the highest lymph node yield, followed by LRC, ORC.

#### Positive surgical margin

Both LRC (OR=-0.25, 95% CI=-0.72 to 0.22) and RARC (OR=-0.05, 95% CI=-0.38 to −0.29) had statistically fewer positive surgical margin rates compared to ORC. Besides, RARC had statistically fewer positive surgical margin rates than LRC (OR=0.22, 95% CI=-0.27 to 0.68) (Figures-7C and D), which can reduce the risk of positive margins.

#### Publication bias

**Figure 8:**
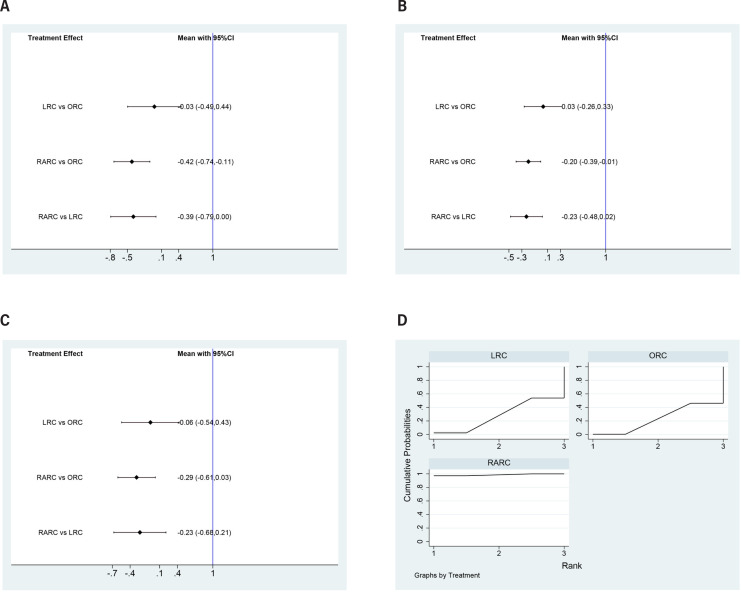
Funnel plot for network meta-analysis of all the outcomes. (A) operative time. (B) overall complication rates.

The publication bias is important for interpreting the conclusions. As shown in Figure-8, the funnel plots had good symmetry, indicating that there had no selectivity and publication bias.

## DISCUSSION

ORC is the "gold standard" for the treatment of MIBC and high-risk NMIBC. However, the surgical procedure is more complicated, time-consuming, and more bleeding (32). With the rapid development of minimally invasive surgical techniques, laparoscopic techniques have been widely used in various urological surgeries, LRC and RARC becoming more and more applied. Parra et al. (54) reported the first LRC in 1992, Menon (55) completed the first RARC in 2003. Compared to LRC, RARC has technological superiorities of better visibility, improved degrees of freedom, and lower learning curves. Despite higher cost and steeper learning curves, minimally invasive surgeries like RARC are being used in many medicine fields (10, 56). According to reports, the proportion of RARC in the United States increased from 0.6% in 2004 to 12.8% in 2010 (57).

In this study, we present an up-to-date network meta-analysis to compare the perioperative and pathological outcomes of RARC, LRC and ORC in bladder cancer. Forty studies were included in our meta-analysis, and the main findings of the present research are as follows: Both LRC and RARC had a longer operative time compared to ORC, no statistically significant difference LRC and RARC. Based on the SUCRA, RARC has the longest surgical time. The amount of blood loss during LRC and RARC was reduced at a statistically significant level compared to ORC, no statistically significant difference LRC and RARC. Based on the SUCRA, LRC has the least bleeding volume. In addition, both LRC and RARC had statistically fewer transfusion rates compared to ORC, RARC had statistically fewer transfusion rates than LRC. No statistically significant difference in hospital stays between LRC and RARC was observed. Based on the SUCRA, LRC has the shortest length of stay. LRC and RARC had significantly shorter time to regular diet than ORC. No statistically significant difference in time to regular diet between LRC and RARC. Based on the SUCRA, RARC has the shortest time to restore normal diet. LRC had significantly shorter time to flatus than ORC. Based on the SUCRA, LRC has the shortest time to flatus. Both LRC and RARC had statistically fewer incidences of overall complications, minor complications, and major complications within 90 days compared to ORC. Besides, RARC had statistically fewer overall complication rates, minor and major complication rates than LRC. LRC, RARC and ORC were comparable in terms of lymph node yield. Both LRC and RARC had statistically fewer positive surgical margin rates compared to ORC. Besides, RARC had statistically fewer positive surgical margin rates than LRC.

The operation time of LRC and RARC is longer than that of ORC because of the complexity of the operation, the high requirements for equipment, and the obvious learning curve. There was no significant difference in surgical time between RARC and LRC. It should be noted that there is no unified standard for surgical time statistics in major medical centers, and robotic surgical systems often require processes such as docking and undocking of operating arms, which may prolong surgical time (49). The actual surgical operation time of RARC may be shorter, but further statistics are needed to determine. In addition, in the early stages of introducing robotic surgery, surgeons and assistants may have a certain learning curve due to lack of experience.

The LRC and RARC surgical incisions are small, which avoids the damage to the skin, muscles and blood vessels caused by the large incisions of ORC surgery, and the intestinal exposure time is short, resulting in less bleeding loss, lower blood transfusion proportion, shorter time to restore normal diet, exhaust time, and hospital stay (58). RARC requires less intraoperative transfusion than LRC, and the amount of intraoperative transfusion required is often determined by intraoperative blood loss and the patient's vital signs.

Both LRC and RARC had statistically fewer incidences of complications than ORC. In addition, the incidence of complications in RARC is the lowest, possibly due to the robot system having a high-definition three-dimensional perspective compared to laparoscopy, allowing surgical operators to distinguish the structure of blood vessels and tissues more clearly and accurately. The seven freely movable robotic arms of the robot can reduce hand tremors while achieving surgical angles that cannot be achieved by laparoscopy. In the narrow space of the pelvic cavity, more precise operations can be performed, reducing errors (42, 59).

Lymph node yield and positive surgical margin status have previously been shown to serve as surrogates for oncologic outcomes. In our network meta-analysis, no significant difference between lymph node yields for LRC, RARC and ORC was observed. Although SUCRA result showed that RARC has the highest lymph node yield, the finding was not significant. The scope of pelvic lymph node dissection under the laparoscope was the same as the open. Due to the magnifying effect of the laparoscope and the clearer field of vision, it can see the lymphatic vessels, swollen lymph nodes, Iliac vessels, obturator nerves, and other important structures to benefit from the complete removal of lymphoid tissues while avoiding neurovascular damage (11). A possible reason for this apparent discrepancy could be the different sampling methods of lymph node collection between the operations. For the robotic groups, at the completion of lymphadenectomy for each side, nodes are submitted as right and left pelvic lymph nodes, whereas in the open group lymph nodes are handed off as discrete anatomical packets (41). The potential risk factors for positive surgical margins are as follows: 1) characteristics of advanced cancer, such as lymphatic vessel invasion, extravesical diseases, and mixed histology; 2) depending on the surgeon's factors, including surgical type, technique, and experience; 3) sample processing. Weihong Xu (60) conducted the first meta-analysis to investigate the effect of surgical margin status on the prognosis of bladder cancer, the findings demonstrate that positive surgical margins were associated with poor outcomes in terms of recurrence-free survival (RFS), cancer-specific survival (CSS) and overall survival (OS) in bladder cancer patients treated with radical cystectomy.

The present study includes some limitations. Firstly, language conditions were set, and data from studies in other languages could not be included. Secondly, the lack of data on some of the study indicators may have an impact on the overall study results.

## CONCLUSIONS

LRC and RARC could be considered as a feasible and safe alternative to ORC for bladder cancer. Notably, compared with LRC, RARC may benefit from significantly lower transfusion rates, fewer complications and lower positive surgical margin rates. These data thus showed that RARC might improve the management of patients with muscle invasive or high-risk non-muscle invasive bladder cancer.
